# Dr. Wm. A. White

**Published:** 1917-12

**Authors:** 


**Dr. Wm. A. White,** (not listed in State Directory), died in Phelps, N. Y., Oct. 14, aged 44.

